# Impact of body position on liver stiffness measurements in men and women with chronic liver disease: liver fibrosis assessed with shear wave elastography comb-push technology

**DOI:** 10.1186/s12880-026-02330-2

**Published:** 2026-04-02

**Authors:** Marie Byenfeldt, Anders Elvin, Per Fransson

**Affiliations:** 1 Clinical Department of Radiology in Östersund, Region Jämtland and Härjedalen, Östersund, Sweden; 2 Department of Health, Medicine, and Caring Sciences, Linköping University, Linköping, Sweden; 3 Center for Medicine Imaging and Visualization Science (CMIV), Linköping University, Linköping, Sweden; 4 Department of Molecular Medicine and Surgery, Karolinska Institute, Stockholm, Sweden; 5 Department of Diagnostics and Intervention, Umeå University, Umeå, Sweden

**Keywords:** Diagnostic techniques and procedures, Ultrasonography, Elasticity imaging technique, Posture, Liver diseases, Obesity, Abdominal, Subcutaneous Fat, Abdominal, Sagittal abdominal diameter, Sex characteristics, Anthropometry

## Abstract

**Background:**

Performing ultrasound shear wave elastography (SWE) in the liver may present difficulties in patients with obesity. The aim of the study was to investigate the impact of different body positions on acoustic radiation force impulse (ARFI) – SWE measurements, skin-to liver capsule distance (SCD) and ARFI-SWE reliability, also stratified for sex.

**Methods:**

In this prospective study, ten ARFI-SWE and ten SCD measurements were obtained from chronic liver disease patients in three body positions: supine, 30° and 90º left decubitus. The study group was stratified into subgroups; increased sagittal abdominal diameter (SAD) (≥ 23 cm) and advanced fibrosis (≥ 9 kPa). Less reliable ARFI-SWE exams were defined as inter-quartile range /median kPa > 30%. Analysis included Friedman’s test, Spearman’s ρ and binary logistic regression.

**Results:**

Analysis of 200 patients showed ARFI-SWE median results 5.5 kPa in supine, 5.8 kPa in 30º and 5.8 kPa in 90º left decubitus (*p* = 0.023). Patients with increased SAD showed no difference in ARFI-SWE results across body positions (*n* = 80; *p* = 0.182), which was seen among patients with no increased SAD (*n* = 120; *p* = 0.020). Among advanced fibrosis cases (*n* = 21), results decreased from 12.2 kPa (supine) to 9.6 kPa (90° left decubitus, *p* = 0.015). The amount of reliable ARFI-SWE exams increased from supine to left decubitus (84.7% -92.6%, *p* = 0.014). The SCD value varied across the supine, 30º and 90º left decubitus body positions (*p* < 0.001; 1.9 cm, 1.8 cm, 1.9 cm, respectively). Only SAD associated with less reliable ARFI-SWE exams (*p* = 0.011; odds ratio = 1.2), and SAD showed a positive correlation to ARFI-SWE result (Spearman’s ρ = 0.515, *p* < 0.001).

**Conclusions:**

SAD appears to influence ARFI-SWE examinations. Adjusting body positioning from supine to left decubitus in patients with higher SAD increases the number of reliable exams and decreases SCD, without increasing ARFI-SWE results, regardless of sex.

## Introduction

In chronic liver disease, fibrosis may arise from various etiologies and can advance to cirrhosis, potentially leading to hepatocellular carcinoma (HCC) [1–5]. The global burden of chronic liver disease remains substantial and continues to increase [6]. Recent literature highlights the growing recognition of the necessity for reliable non-invasive methods to detect, monitor, and stage liver fibrosis, particularly for identifying compensated cirrhosis [7, 8].

Studies have demonstrated that the non-invasive 2D shear wave elastography (SWE) technology using push pulses termed acoustic radiation force impulse (ARFI) measuring liver stiffness, have shown an excellent correlation with the results of liver biopsy in the assessment of fibrosis [9, 10]. ARFI-SWE is regarded as a rapid and cost-effective way to monitor, detect, and stage liver fibrosis in patients with liver diseases [10, 11]. The performance of ARFI-SWE is outlined in SWE guidelines [12–14], where a reliable ARFI-SWE examination is defined by a median kPa derived from 5 to 10 measurements with an interquartile range interval (IQR) /median kPa ≤ 30%, ARFI-SWE results not meeting this criterion are considered less reliable ARFI-SWE examination. Moreover, ongoing education in ultrasound practice is essential to uphold patient safety and maintain high standards in diagnostic procedures [15, 16], and it is critical to recognize and appropriately manage factors that may adversely affect ARFI-SWE diagnostic performance and reliability [17–21]. Optimizing the handling of these factors will enhance the diagnostic performance of ARFI-SWE and promote its broader clinical application [22].

The prevalence of overweight and obesity in both adults and children has been steadily increasing; between 1990 and 2022, adult obesity rates have more than doubled from 7% to 16% [23]. Corresponding with this rise [23], clinicians are encountering more patients with increased central adiposity. Central adiposity refers to visceral and subcutaneous fat accumulation in the abdominal region, which is a recognized risk factor for chronic cardiometabolic diseases [24]. Visceral adiposity can be assessed through anthropometric measures such as sagittal abdominal diameter (SAD) [25–27], which has previously been studied as a predictor for severe liver disease [28], and a SAD cut-off value of 23 cm has been identified as linked to metabolic syndrome [25]. Elevated intra-abdominal pressure (IAP) is associated with obesity [29] and increased SAD [30, 31].

Previous studies have reported morphological and hemodynamic changes in liver-associated vasculatures including the inferior vena cava [32, 33], portal vein [34] and abdominal aorta [32] in response to altered body positions. Cardiac insufficiency has been identified as a hemodynamic factor that can increase liver stiffness regardless of fibrosis stage [35], and increased arterial pressure has also been noted to contribute to higher liver stiffness [36]. Following transjugular intrahepatic portosystemic shunt (TIPS) implantation, a reduction of liver stiffness was observed [37]. Furthermore, treatment with cardiovascular medication appears to enhance the correlation between liver stiffness and portal vein pressure [38].

ARFI-SWE liver assessments have demonstrated significant differences in measurements between patients positioned upright and those in supine positions [39, 40]. Furthermore, studies involving healthy individuals have indicated that ARFI-SWE results are elevated in the left decubitus and standing positions compared to the supine body position [39, 41–43]. These body positional variations in liver stiffness are likely attributable to impaired venous drainage and intrahepatic blood stasis.

Subcutaneous adiposity may be assessed by measuring the skin-to liver capsule distance (SCD) and prior studies have demonstrated that the thickness of the abdominal can influence ARFI-SWE results [44–48]. A reduction in SCD enhances B-mode image quality, improves signal-to noise ratio (SNR), and increases shear wave quality in the elastogram map. Notably, research has indicated that increased probe force, which decreases SCD, correlates with a higher technical success rate in ARFI-SWE examinations. However, when using increased probe force to decrease SCD, increased SAD remains associated with less reliable ARFI-SWE examinations in men, but not in women [49]. Women generally exhibit less visceral adipose tissue and more subcutaneous fat compared to men, who possess higher amounts of intra-abdominal fat [50, 51]. Sex-based differences are recognized as significant factors in various diseases, such as abdominal obesity measures for cardiovascular disease risk [52], and these differences also manifest in liver fibrosis [42, 53–57]. Among non-invasive diagnostic tests, the Fatty Liver Index has exhibited a 50% variance between sexes, underscoring the necessity for sex-specific, personalized screening and prevention programs for dysmetabolism-related liver conditions [58, 59].

It is essential to identify confounding factors to ensure the accurate interpretation of liver stiffness in clinical practice. Since body position affects hepatic hemodynamics, it may significantly influence liver stiffness measurements. Considering established anatomical and pathophysiological differences between sexes, along with the limited research on the causal relationship between body position and liver stiffness, including variables such as SAD and SCD that could impact ARFI-SWE results across various body positions, we hypothesized that liver stiffness values are likely to vary with changes in body position.

The aim of the study was to investigate the impact of different body positions on ARFI-SWE liver stiffness measurements, SCD and ARFI-SWE reliability in a cohort of patients with chronic liver disease, also stratified for sex.

## Material and methods

### Study population

**Fig. 1:**
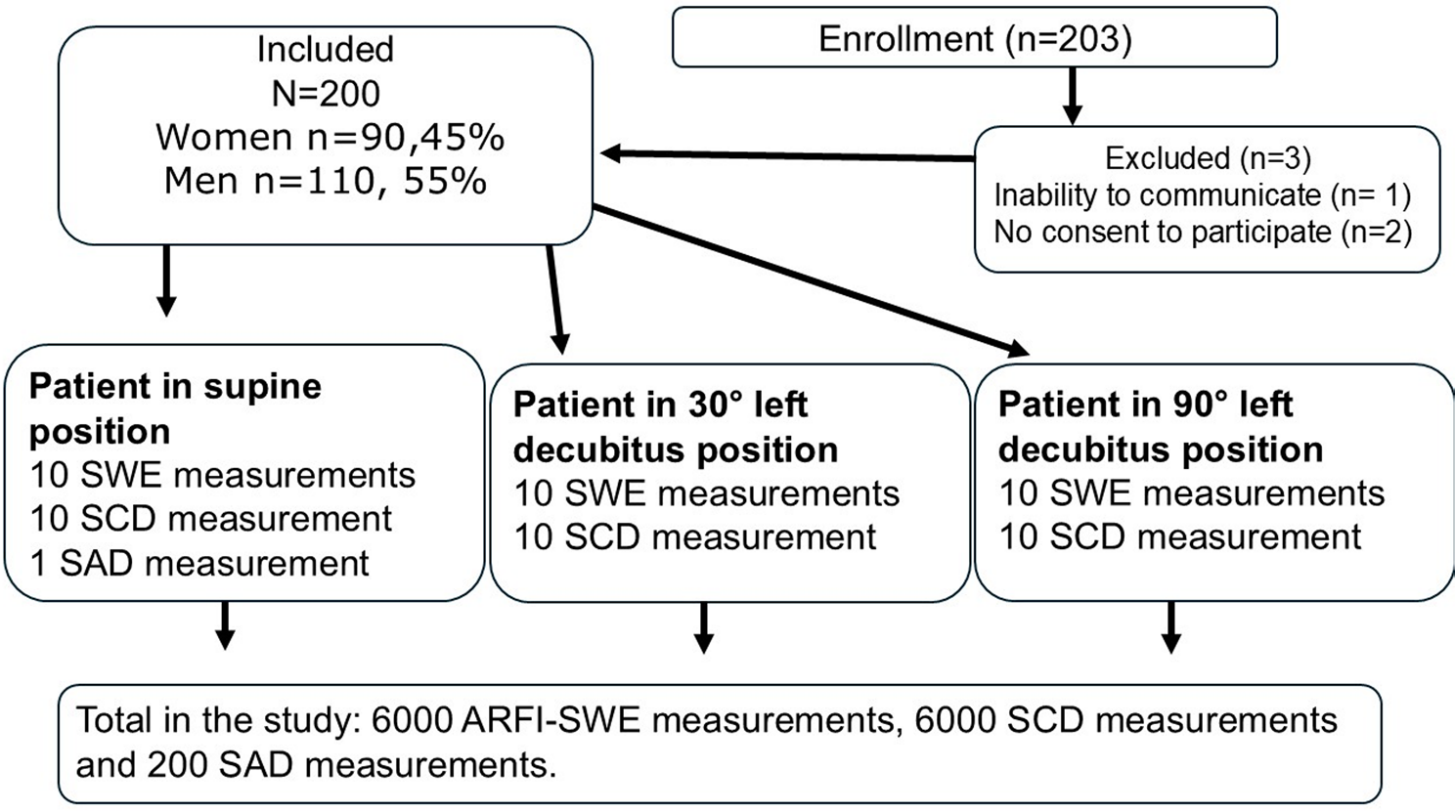
Flowchart depicting the process of patient selection and measurement protocols utilized in the study. Each participant functioned as their own control. SWE refers to shear wave elastography, ARFI to acoustic radiation force impulse, SCD to skin-to-liver capsule distance and SAD to sagittal abdominal diameter

This single-center, cross-sectional clinical study prospectively enrolled 203 consecutive patients diagnosed with liver disease who were referred to the radiology department for ARFI-SWE between August 2017 and May 2018. Three patients were excluded due to inability to communicate (*n* = 1) or no consent to participate (*n* = 2). Thus, this study included 200 patients who gave their written informed consent, as detailed in Table 1; Fig. 1. The study received approval from the Research Ethical Review Board in Umeå, Sweden (Nos: 2017-78-31, 2017-417-32 M, and 2017-302-32 M), and was conducted in accordance with the World Medical Association Declaration of Helsinki (2013). The study collected data regarding patients’ use of cardiovascular medications (ATC codes: C07 AB03, C07 AA05, C07 AB07, C07 AG02, C07 AB02, C07 AB02, C07 AA07) as well as the presence of type 2 diabetes.

### Ultrasound measurements

Liver stiffness measurements were obtained with the GE LOGIQ E9 ultrasound device revision 2 (General Electric Healthcare, Milwaukee, Wisconsin, USA), with a convex probe (C1–6), and the ARFI-SWE comb-push technology. ARFI-SWE measurements were performed according to the EFSUMB guidelines [11], and in addition to supine body position also with 30° left decubitus and 90º left decubitus body positions.

A fixed ROI with a 1.25-cm diameter, and an area (A = πd^2^/4) of 1.23 cm^2^ was selected. All measurements were performed within the same liver segment in all body positions, and ROI was placed with an angle of the beam close to zero. The median kPa result of 10 ARFI-SWE measurements were used for analysis. Patients unable to independently hold their breath were provided with a nose clip and instructed to close their mouth to suspend breathing during measurements.

Directly after ARFI-SWE examination on the same day, the presence of hepatic steatosis was evaluated using GE LOGIQ S8 XDclear 2.0 ultrasound device revision 4 (General Electric Healthcare, Seoul, Korea) with integrated controlled attenuation parameter (CAP) program, equipped with M and XL probes calibrated by the manufacturer. Ten CAP measurements were performed in each patient in supine position, generating mean CAP value used to rule out (< 248 dB/m) or confirm (≥ 248 dB/m) the presence of hepatic steatosis ≥ S1, according to latest publication from EASL-EASD-EASO Clinical Practice Guidelines on the management of metabolic dysfunction-associated steatotic liver disease (MASLD) [5, 60].

The presence of ascites was confirmed or ruled out using standard B-mode ultrasound examination at same time as ARFI-SWE examination. The cirrhosis diagnosis in the study population in Table 1 was clinically determined.

All patients had fasted for at least 5 h and rested for a minimum of 10 min before examination. All measurements in the study for each patient were performed on the same day.

The ultrasound operator possessed five years of clinical experience and completed EFSUMB-accredited training in shear wave elastography of the liver across multiple ultrasound platforms.

### Anthropometric measurements

**Fig. 2:**
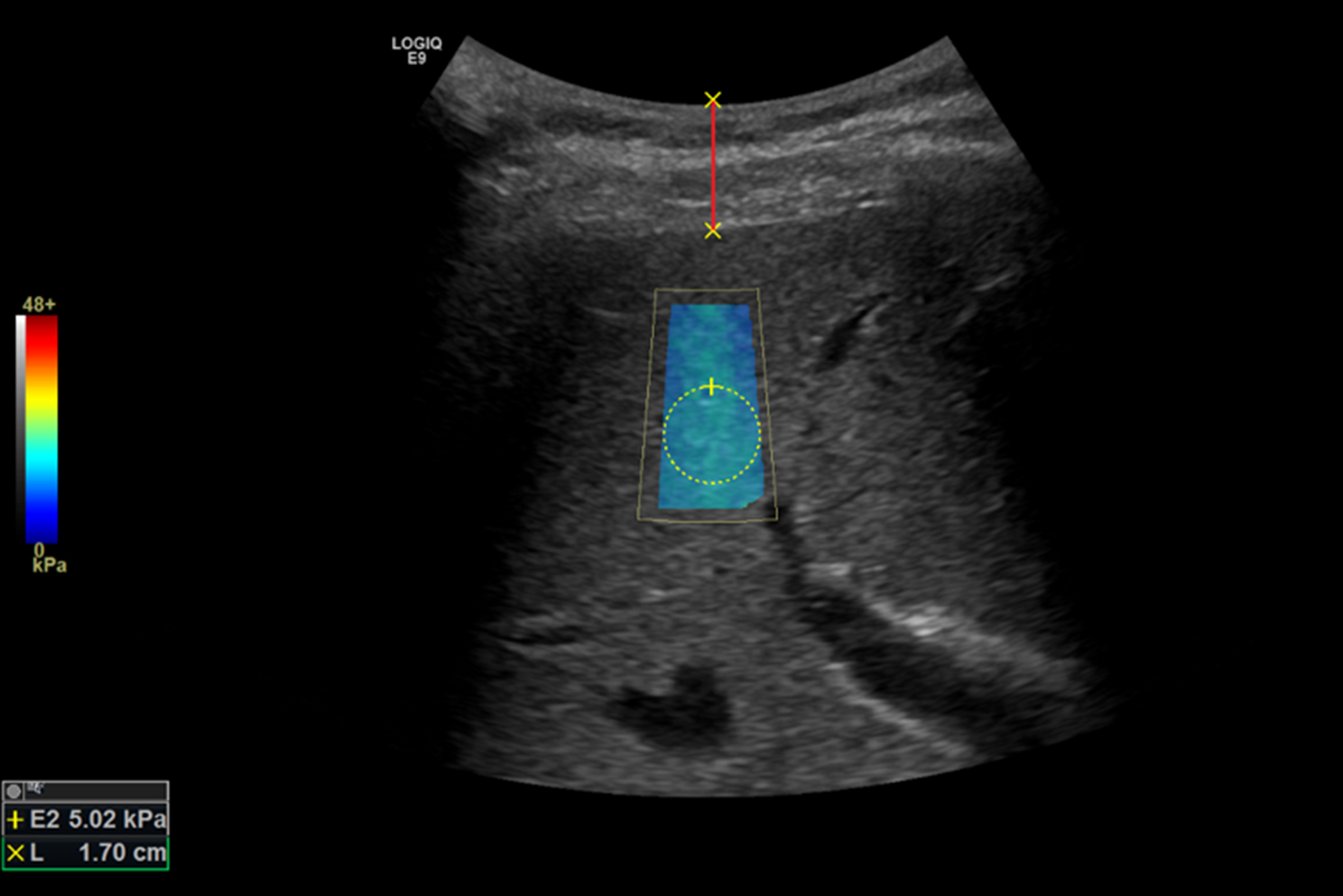
Ultrasound image: The fully color-filled shear wave elastography (SWE) and the homogeneous elastogram sample box indicate technical success and high-quality shear wave acquisition. The circle denotes the region of interest (ROI; spherical volume), while the red line represents the skin-to-liver capsule distance (SCD)

SCD was defined as the distance from the skin surface to the liver capsule, utilizing the same images employed for ARFI-SWE measurement (see Fig. 2). For each patient, ten SCD measurements were obtained from each body position.

SAD was defined as the distance between the firm examination table up to the horizontal level with patient body in supine position and measured after a normal expiration to nearest 0.5 cm, with straight legs. One SAD measurement was sampled in each patient in supine position at the umbilical level, without clothes in the measurement area, using a ruler and water level [27]. SAD value of ≥ 23 cm was defined as increased [25], and hypothesized in this study to have impact on liver stiffness.

Body mass index (BMI) was calculated from weight and height measurements obtained on site with the patients wearing light clothing and no shoes.

### Body positions

Ten ARFI-SWE measurements were performed for each patient across three sequential body positions: supine, 30° left decubitus, and 90º left decubitus. All measurements were taken from identical liver segments at consistent depths and conducted on the same day. For the 30° left decubitus position, a custom-designed pillow was used to maintain uniformity in patient positioning.

### Definition of ARFI-SWE reliability

ARFI-SWE reliability was expressed according to SWE liver guidelines definition of reliable ARFI-SWE examinations, median kPa of ten measurements with IQR /median kPa > 30% for less reliable examinations [12–14]. Accordingly, reliable ARFI-SWE examinations in this study were defined as IQR/median kPa ≤ 30% of ten measurements.

### Definition of advanced fibrosis

Current SWE guidelines from WFUMB recommend that, irrespective of the ultrasound system used, an ARFI-SWE value of ≥ 9 kPa should be applied to identify advanced liver fibrosis in patients with chronic liver disease [61]. This threshold is utilized in the present study to categorize subjects into groups with advanced fibrosis and those without advanced fibrosis.

### Statistical analysis

Descriptive statistics were produced for demographic characteristics. Liver stiffness measurements were expressed in Young´s modulus kPa, using ARFI-SWE examination of ten measurements for ARFI-SWE median kPa result with IQR. Continuous variables are expressed as the mean ± standard deviation (min-max), median (min-max). Categorical variables are expressed as the frequency and percentage. The normality of the continuous data was tested by the one-sample Kolmogorov–Smirnov test. Body mass index (BMI) was calculated as weight (kg) divided by height (m) squared. Each patient served as his/her own control in repeated ARFI-SWE and SCD measurements in the three different body positions.

To assess the impact of the three body position groups on ARFI-SWE results, as well as the SCD and ARFI-SWE reliability, the Friedman’s test, Wilcoxon signed-rank test, and McNemar’s test were employed, with analyses additionally stratified by sex.

Spearman’s ρ was utilized to assess the relationship between SCD, SAD, and ARFI-SWE results, with analyses also stratified by sex.

To assess the independent impact of cardiovascular medication, sex, SCD, and SAD on the reliability of ARFI-SWE examinations, binary logistic stepwise regression analyses were conducted to determine their association with less reliable ARFI-SWE results. Significant factors from the univariate analyses were then analyzed in a multiple logistic regression model, also stratified for sex. Nagelkerke R² and Cox & Snell R² were used to evaluate the model. The agreement between paired readings of median kPa ARFI-SWE results log10 obtained from the three different body positions were illustrated with Bland -Altman plots [62].

The p-value of < 0.05 was considered significant and all statistical tests were two-sided. Statistical analyses were performed in the software Statistical Package for Social Sciences (SPSS) version 31.

## Results

Within the study population, 108 out of 200 participants (53.2%) were infected with hepatitis C virus, 53 (26.1%) were infected with hepatitis B virus, and 12 (5.9%) were clinically diagnosed with metabolic dysfunction-associated steatotic liver disease (MASLD). No cases of ascites were detected among the patients. A detailed overview of participant characteristics is provided in Table 1.

For this investigation, a total of 6000 ARFI-SWE measurements, 6000 SCD measurements, and 200 SAD measurements were obtained from the 200 patients, as illustrated in Fig. 1.

**Table 1:** Demographic and clinical profile of the study population

*N* = 200	
**Age** years, mean (± SD)	46.7 (15.5)
**Sex**, n (%)	
Women	90 (45)
Men	110 (55)
**Body Mass Index**, kg/m², mean (± SD)	27.3 (5.3)
**Skin-to-liver capsule distance (SCD)**, cm, median (min-max)	
Supine position	1.9 (1.0-3.9)
**Sagittal abdominal diameter (SAD)**, cm, median (min-max)	21.5 (15.0-39.0)
**Hepatic steatosis** n (%)	
No steatosis	113 (56.5)
Steatosis, ≥ S1	87 (43.5)
**Cardiovascular medication**	
No intake of medication	160 (80.0)
Intake of medication	40 (20.0)
**Cirrhosis** n (%)	
No cirrhosis	191 (95.5)
Cirrhosis	9 (4.5)
**Type 2 Diabetes** n (%)	
No diabetes	187 (93.5)
Diabetes	13 (6.5)

**Fig. 3:**
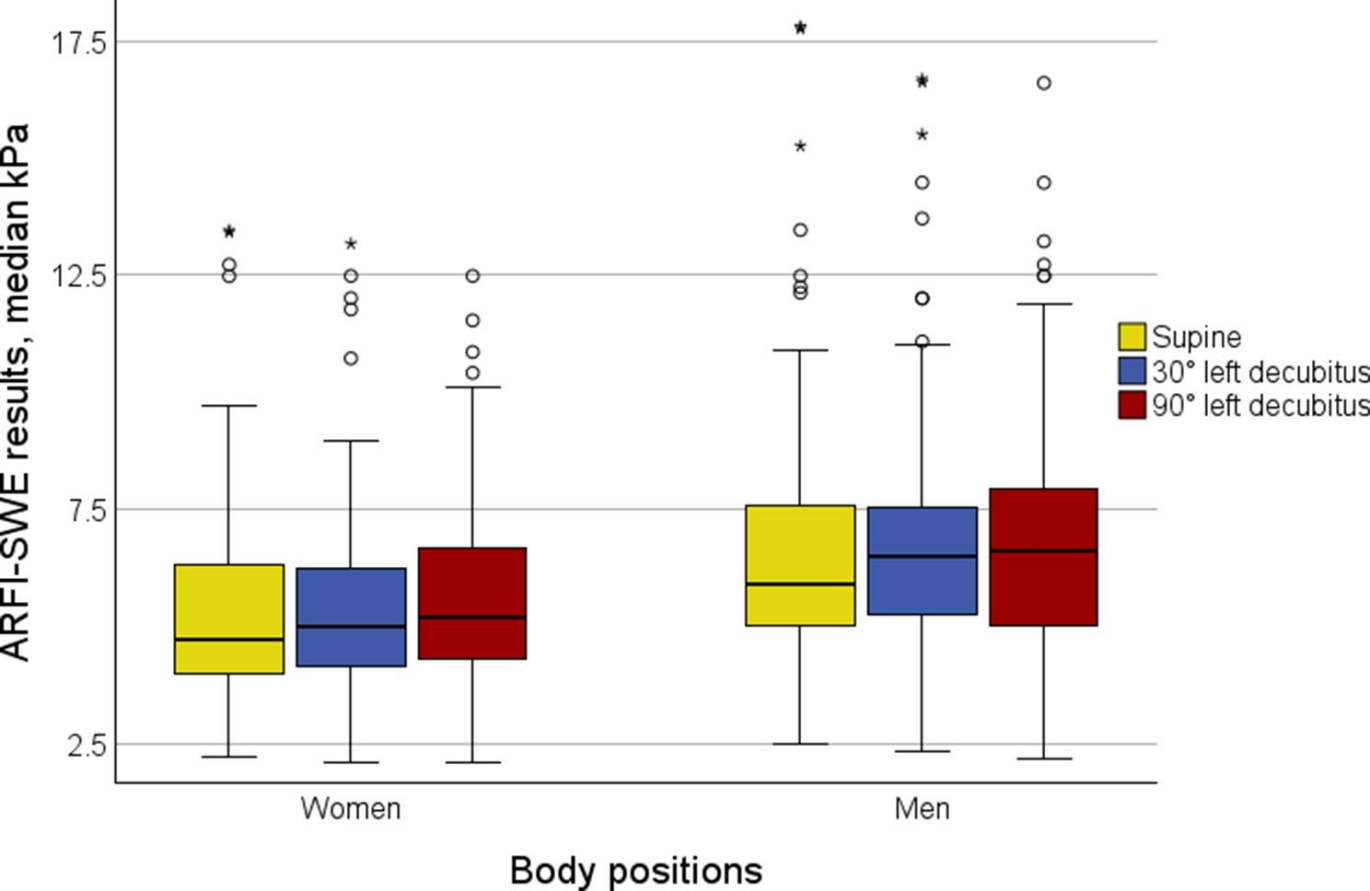
Median kPa acoustic radiation force impulse (ARFI) - shear wave elastography (SWE) results for the supine, 30° left decubitus and 90° left decubitus body positions stratified for sex. Boxplots show the median, the two central quartiles in the box, one quartile in each whisker, and outliers. The ARFI-SWE results were in the supine position 5.5 kPa (women 4.7 kPa, men 5.9 kPa), in 30º left decubitus 5.8 kPa (women 5.0 kPa, men 6.5 kPa), and in 90º left decubitus 5.8 kPa (women 5.2 kPa, men 6.6 kPa). Differences across body positions were seen in the study group (*N* = 200; *p* = 0.023) and among women (*n* = 90; *p* = 0.038), but not among men (*n* = 110; *p* = 0.296)

The outcomes for the study group are summarized in Table 2 and illustrated in Fig. 3. Findings specific to the SAD subgroup are provided in Table 3, while findings for the advanced fibrosis subgroup are detailed in Table 4. Additionally, data for men and women are reported separately across all tables.

### Body positions impact on ARFI-SWE result, SCD and ARFI-SWE reliability

The ARFI-SWE results in the supine position were 5.5 kPa overall (women 4.7 kPa, men 5.9 kPa), in the 30º left decubitus position, results were 5.8 kPa (women 5.0 kPa, men 6.5 kPa), and in the 90º left decubitus positions, values remained at 5.8 kPa (women 5.2 kPa, men 6.6 kPa), as detailed in Table 2. Differences were seen across body positions in the study population (*N* = 200; *p* = 0.023) and among women (*n* = 90; *p* = 0.038) but not among men (*n* = 110; *p* = 0.296), Table 2.

Further sex-stratified analysis indicated no differences in ARFI-SWE results across body positions: supine vs. 30º left decubitus (men *p* = 0.089, women *p* = 0.260); supine vs. 90º left decubitus (men *p* = 0.176, women *p* = 0.065); and 30º vs. 90º left decubitus (men *p* = 0.668, women *p* = 0.253). Figure 3 illustrates the ARFI-SWE results obtained from the three body positions stratified for sex.

The SCD values were significantly different among the supine (1.9 cm), 30º left decubitus (1.8 cm), and 90º left decubitus (1.9 cm) positions (*p* = 0.005; 1.9 cm, 1.8 cm, 1.9 cm, respectively), Table 2. In further analysis the difference was found between supine body position and 30º left decubitus (*p* < 0.001; 1.9 cm vs. 1.8 cm).

Further sex-based analysis for SCD measurements across body positions revealed differences between the supine vs. 30º left decubitus positions for men (*n* = 110; *p* = 0.002), and for women, between supine vs. 30º left decubitus (*n* = 90; *p* = 0.003), as well as between 30º left decubitus vs. 90º left decubitus (*n* = 90; *p* = 0.012).

Interestingly, maximum observed SCD value for one patient was 3.9 cm in the supine position, and this SCD value decreased to 3.0 cm at 30º left decubitus and further to 2.9 cm at 90º left decubitus. This indicates a reduction of 1 cm in SCD with the change from supine to 90º left decubitus position for this single patient.

**Table 2:** Body positions impact on ARFI-SWE results, SCD and the ARFI-SWE reliability

Body positions impact on ARFI-SWE results, SCD and ARFI-SWE reliability	Supine	30° left decubitus	90° left decubitus	*p*-value*
ARFI-SWE result, median kPa (min-max)	5.5 (2.2–17.8)	5.8 (2.1–16.7)	5.8 (2.1–16.6)	0.023
women	4.7 (2.2–13.4)	5.0 (2.1–13.2)	5.2 (2.1–12.5)	0.038
men	5.9 (2.5–17.8)	6.5 (2.3–16.7)	6.6 (2.2–16.6)	0.296
SCD, median cm (min-max)	1.9 (1.0–3.0)	1.8 (1.1–3.4)	1.9 (1.1–3.4)	0.005
women	1.8 (1.2–3.9)	1.7 (1.0-3.1)	1.8 (1.1–3.1)	0.221
men	2.0 (1.0-3.8)	1.9 (1.0-3.7)	1.9 (1.1–3.4)	0.007
ARFI-SWE reliability; Reliable, n /Less reliable, n	172/28	175/25	188/12	0.020
women	78/12	78/12	80/10	0.916
men	94/16	97/13	108/2	0.002

*Friedman´s test. ARFI refers to acoustic radiation force impulse, SWE to shear wave elastography and SCD to skin-to-liver capsule distance

In supine body position, there was a positive correlation between ARFI-SWE result and SCD measurements (*N* = 200, Spearman’s ρ = 0.426, *p* < 0.001), for men (*n* = 110, Spearman’s ρ = 0.337, *p* < 0.001) and for women (*n* = 90, Spearman’s ρ = 0.521, *p* < 0.001). In the 30º left decubitus position, there was a positive correlation between ARFI-SWE result and SCD measurements (*N* = 200, Spearman’s ρ = 0.362, *p* < 0.001), for men (*n* = 110, Spearman’s ρ = 0.291, *p* < 0.001) and for women (*n* = 90, Spearman’s ρ = 0.323, *p* < 0.001). In the 90º left decubitus position, there was a positive correlation between ARFI-SWE result and SCD measurements (*N* = 200, Spearman’s ρ = 0.455, *p* < 0.001), for men (*n* = 110, Spearman’s ρ = 0.458, *p* < 0.001) and for women (*n* = 90, Spearman’s ρ = 0.440, *p* < 0.001).

A total of 172 (84.7%) reliable ARFI-SWE examinations were obtained in the supine position, 175 (86.2%) in the 30° left decubitus position, and 188 (92.6%) in the 90° left decubitus position. Analysis of the number of reliable ARFI-SWE examinations across different body positions indicated significant differences exclusively among men, specifically between supine vs. 30° left decubitus (*n* = 94 vs. *n* = 97; *p* < 0.001) and between 30° vs. 90° left decubitus (*n* = 97 vs. *n* = 108; *p* = 0.007). No significant differences were observed among female patients (N.S).

### Agreement and bias in ARFI-SWE results across body positions: bland-altman analysis

The mean kPa difference between the supine position vs. the 90° left decubitus was statistically significant (*p* < 0.001), making Bland-Altman analysis unsuitable for these two body positions. Conversely, Bland-Altman analysis was applicable to the comparison between the supine vs. 30° left decubitus positions (N.S).

**Fig. 4:**
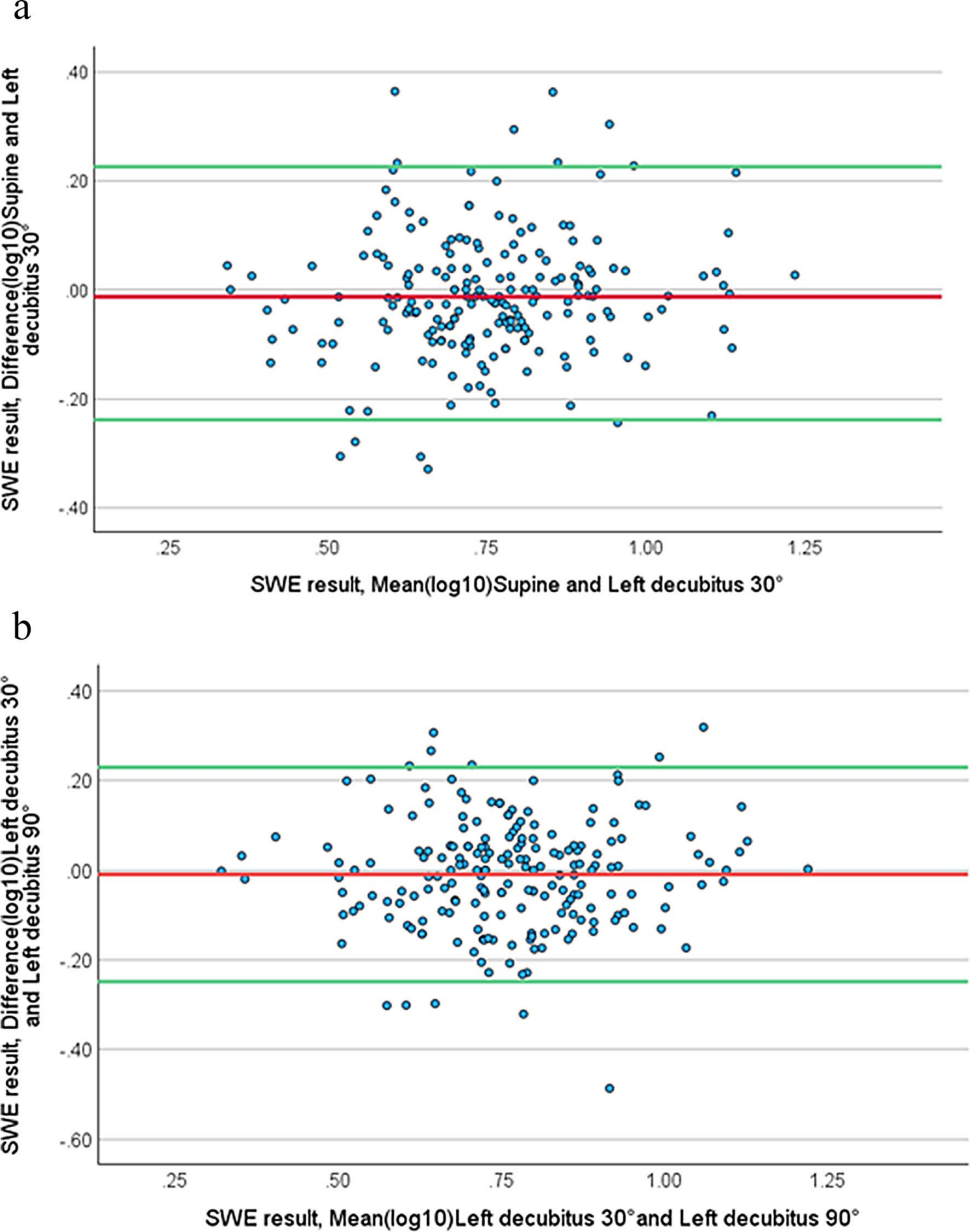
**a** Bland-Altman diagram for supine vs. 30° left decubitus body position. The average for log10 kPa in supine and 30° left decubitus is plotted on the x-axis, and the difference (log10 kPa in supine subtracted from log 10 kPa in 30° left decubitus body position) is shown on the y-axis. The central red line indicates the mean difference of − 0.013. Green lines indicate the 95% confidence intervals limits of agreement (LOA). SWE refers to shear wave elastography, created with acoustic radiation force impulse (ARFI). **b**. Bland-Altman diagram for 30° left decubitus vs. 90° left decubitus body position. The average for log10 kPa in 30° left decubitus and 90° left decubitus body position is plotted on the x-axis, and the difference (log10 kPa in 30° left decubitus subtracted from log10 kPa in 90° left decubitus body position) is shown on the y-axis. The central red line indicates the mean difference of − 0.0096. Green lines indicate the 95% confidence intervals limits of agreement (LOA). SWE refers to shear wave elastography, created with acoustic radiation force impulse (ARFI)

The mean log10 kPa differences between the supine position vs. 30° left decubitus (-0.013) are illustrated in the Bland-Altman diagram. The plots are 95% within the limits of agreement (LOA) and subjectively assessed to be randomly distributed without signs of proportional bias (β = 0.060; *p* = 0.173), as shown in Fig. 4a.

Similarly, the mean log10 kPa differences between the 30° and 90° left decubitus positions (-0.0096) are illustrated in the Bland-Altman diagram. Here, 95% of the plots are within LOA and are subjectively assessed to be randomly distributed, with no indication of proportional bias (β = 0.028; *p* = 0.625), as depicted in Fig. 4b.

A small number of plots that lie outside the 95% LOA can be observed in the mid-range of mean log10 ARFI-SWE kPa on both Bland-Altman diagrams.

### Impact of body position on ARFI-SWE result, SCD and ARFI-SWE reliability in subgroups defined by sagittal abdominal diameter

In the subgroup of patients without increased SAD (*n* = 120), a difference in ARFI-SWE results was observed between body positions (*p* = 0.020; see Table 3). The difference was found between the supine position and the 30° left decubitus position (5.0 kPa vs. 5.3 kPa; *p* < 0.001).

Moreover, further sex-stratified analysis among men in this subgroup the difference was noted between the supine vs. 30° left decubitus positions (*n* = 59; 5.5 kPa vs. 5.9 kPa, *p* = 0.029). For women, differences were identified between supine vs. 30° left decubitus (*n* = 61; 4.6 kPa vs. 4.7 kPa, *p* = 0.015), as well as between supine vs. 90° left decubitus (*n* = 61; 4.6 kPa vs. 5.0 kPa, *p* = 0.012).

Among patients without increased SAD, SCD values were highest in the 90° left decubitus position. In women, a difference was found between the 30° vs. 90° left decubitus positions (*n* = 120; 1.5 cm vs. 1.7 cm, *p* < 0.001), while no differences were observed among men (N.S).

Within patients without increased SAD, the supine position resulted in the highest number of reliable examinations (*n* = 133); however, there were no differences of reliable examinations between body positions overall (N.S). Among men, a notable difference was detected between the 30° vs. 90° left decubitus positions (*n* = 49 vs. *n* = 59, *p* = 0.002), whereas no differences were found among women (N.S).

In patients with increased SAD (*n* = 80), no differences in ARFI-SWE results were observed across the three body positions, irrespective of sex, Table 3 (N.S).

SCD measurements were lower in both the 30° and 90° left decubitus positions compared to supine; specifically, differences were noted between supine vs. 30° left decubitus (*n* = 80, 2.3 cm vs. 2.2 cm, *p* < 0.001) as well as between supine vs. 90° left decubitus (*n* = 80, 2.3 cm vs. 2.2 cm, *p* = 0.003).

The highest number of reliable examinations within the increased SAD subgroup was achieved in the 90° left decubitus position (*n* = 76, *p* < 0.001).

Further analysis by sex revealed that, among men, there were differences in the number of reliable examinations between supine vs. 30° left decubitus (*n* = 40 vs. *n* = 48, *p* = 0.021) and between supine vs. 90° left decubitus (*n* = 40 vs. *n* = 49, *p* = 0.012), with the highest number of reliable examinations observed in the 90° left decubitus position. Among women, a difference was identified between supine vs. 90° left decubitus positions (*n* = 19 vs. *n* = 27, *p* = 0.039), again with the highest number of reliable examinations in the 90° left decubitus position.

**Table 3:** Body position impact on ARFI-SWE results, SCD, and the ARFI-SWE reliability in subgroups of sagittal abdominal diameter (SAD), also stratified for sex

Body positions impact on ARFI-SWE results, SCD and ARFI-SWE reliability in subgroups defined by sagittal abdominal diameter. *N* = 200	Supine	30° left decubitus	90° left decubitus	*p*-value*
**SAD < 23 cm (n = 120)**				
ARFI-SWE results, median kPa (min-max)	5.0 (2.2–12.7)	5.3 (2.1–15.5)	5.2 (2.1–11.2)	0.020
women, *n* = 61	4.6 (2.2–12.7)	4.7 (2.1–12.0)	5.0 (2.1–10.1)	0.013
men, *n* = 59	5.5 (2.5–12.1)	5.9 (2.3–15.5)	5.7 (2.2–11.2)	0.137
SCD, median cm (min-max)	1.6 (1.0-3.3)	1.6 (1.0-2.7)	1.7(1.1–2.9)	0.052
women, *n* = 61	1.6 (1.2–2.8)	1.5 (1.1–2.7)	1.6 (1.1–2.9)	0.009
men, *n* = 59	1.8 (1.0-3.3)	1.7 (1.0-2.4)	1.7 (1.1–2.6)	0.509
ARFI-SWE reliability; Reliable, n /Less reliable, n	133/7	104/16	112/8	0.081
women, *n* = 61	59/2	55/5	53/8	0.155
men, *n* = 59	54/5	49/10	59/0	0.005
**SAD ≥ 23 cm (n = 80)**				
ARFI-SWE result, median kPa (min-max)	6.8 (3.6–17.8)	6.6 (3.2–16.7)	7.3 (2.8–16.6)	0.182
women, *n* = 29	6.8 (3.6–13.4)	5.3 (3.7–13.2)	6.0 (2.8–12.5)	0.764
men, *n* = 51	6.8 (4.1–17.8)	6.8 (3.2–16.7)	7.8 (4.2–16.6)	0.175
SCD, median cm (min-max)	2.3 (1.5–3.9)	2.2 (1.3–3.7)	2.2 (1.3–3.4)	< 0.001
women, *n* = 29	2.3 (1.3–3.9)	2.4 (1.6–3.1)	2.2 (1.6–3.1)	0.024
men, *n* = 51	2.2 (1.5–3.8)	2.1 (1.3–3.7)	2.1 (1.3–3.4)	0.003
ARFI-SWE reliability; Reliable, n /Less reliable, n	59/21	71/9	76/4	< 0.001
women, *n* = 29	19/10	23/6	27/2	0.025
men, *n* = 51	40/11	48/3	49/2	0.004

*Friedman´s test. SAD refers to sagittal abdominal diameter, SCD to skin-to-liver capsule distance, SWE to shear wave elastography and ARFI to acoustic radiation force impulse

**Fig. 5:**
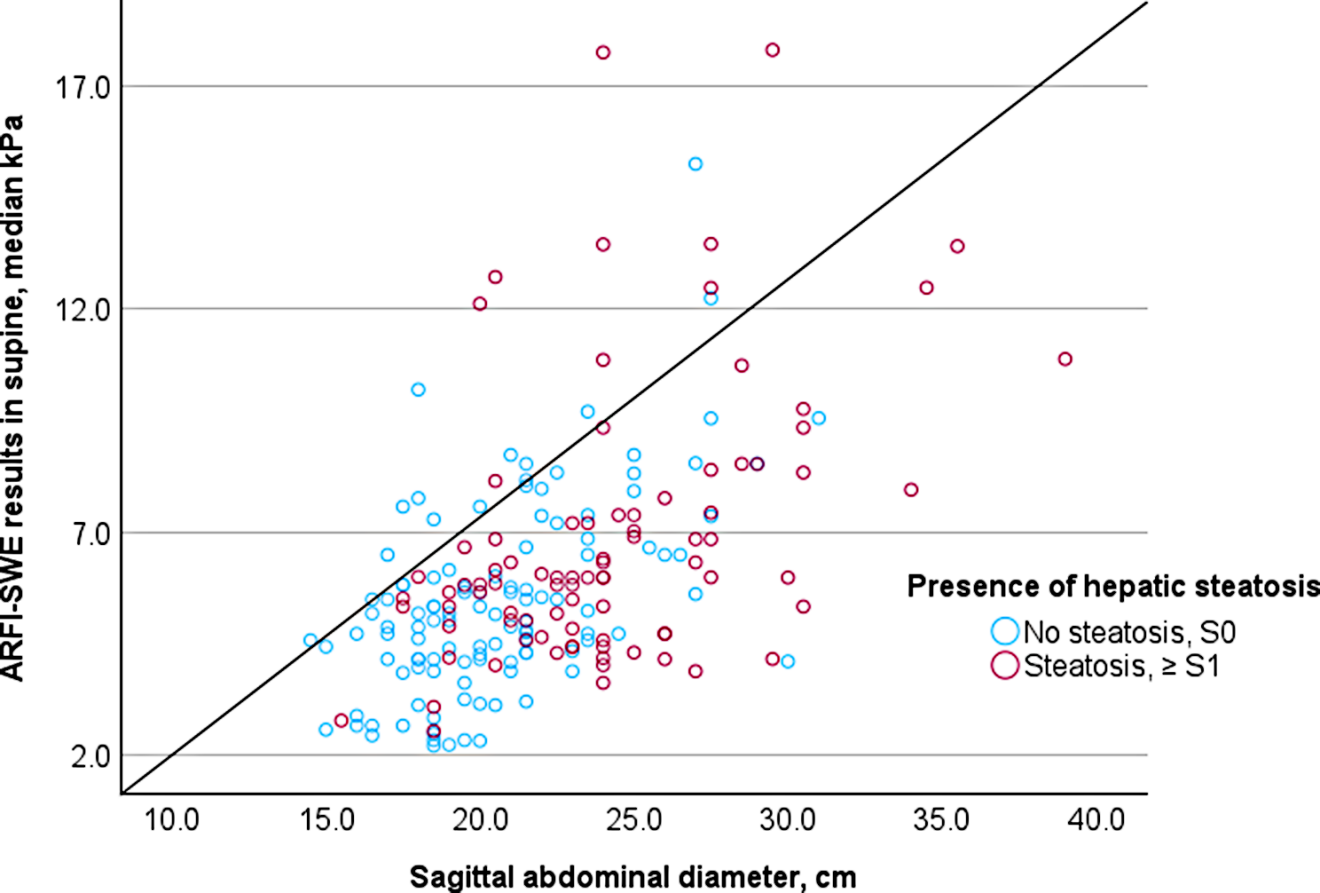
There was a positive correlation between sagittal abdominal diameter (SAD) and shear wave elastography (SWE) results (*N* = 200, Spearman’s ρ = 0.515, *p* < 0.001). SWE was created with acoustic radiation force impulse (ARFI)

In the supine position, a positive correlation was observed between ARFI-SWE results and SAD (*N* = 200; Spearman’s ρ = 0.515, *p* < 0.001), as shown in Fig. 5, both among men (*n* = 110; Spearman’s ρ = 0.416, *p* < 0.001) and women (*n* = 90; Spearman’s ρ = 0.489, *p* < 0.001).

Within the subgroup without increased SAD, there was a positive correlation between ARFI-SWE results and SAD (*n* = 120; Spearman’s ρ = 0.332, *p* < 0.001). However, when stratified by sex, no significant correlations were observed for either men or women.

For patients with increased SAD, a positive correlation between ARFI-SWE results and SAD was found (*n* = 80; Spearman’s ρ = 0.448, *p* < 0.001), which remained significant in men (*n* = 51; Spearman’s ρ = 0.527, *p* < 0.001) and also in women (*n* = 29; Spearman’s ρ = 0.375, *p* = 0.045).

### Impact of body position on ARFI-SWE results in subgroups defined by advanced fibrosis

In the subgroup of patients with advanced fibrosis determined by ARFI-SWE in the supine position (*n* = 21), the median SAD was 27.3 cm, compared to 21.6 cm in patients without advanced fibrosis (*n* = 179; *p* < 0.001, see Table 4). Among patients without advanced fibrosis, liver stiffness increased from 5.3 kPa in the supine position to 5.6 kPa at 90° left decubitus (*n* = 179; *p* < 0.001, see Table 4). Conversely, in those with advanced fibrosis, liver stiffness decreased from 9.3 kPa supine to 5.0 kPa at 90° left decubitus (*n* = 21; *p* = 0.015, see Table 4).

Sex-specific differences were identified among patients without advanced fibrosis, as women demonstrated an increase in liver stiffness, absent in men, when body position changed from supine to left decubitus. Given the small sample size of cases with advanced fibrosis, a sex-based analysis was not conducted.

**Table 4:** Body position impact on liver stiffness measurements in subgroup of advanced fibrosis ≥ 9 kPa, also stratified for sex

Body positions impact on liver stiffness in subgroups defined by advanced fibrosis. *N* = 200	Supine	30° left decubitus	90° left decubitus	* p*-value*
**No advanced fibrosis (n = 179) **				
ARFI-SWE results, median kPa (min-max)	5.3 (2.2–8.7)	5.5 (2.1–12.0)	5.6 (2.1–13.2)	< 0.001
women *n* = 83	4.6 (2.2–8.7)	4.7 (2.1–11.8)	5.2 (2.1–10.9)	0.003
men *n* = 96	5.6 (2.5–8.7)	6.3 (2.3–12.0)	6.2 (2.2–13.2)	0.123
**Advanced fibrosis (n = 21)**				
ARFI-SWE results, median kPa (min-max)	12.2 (9.3–17.8)	11.0 (4.7–16.7)	9.6 (5.0-16.6)	0.015
women *n* = 7	12.5 (9.3–13.5)	10.7 (6.2–13.2)	7.7 (5.9–12.5)	-
men *n* = 14	11.5 (9.3–17.8)	11.1 (4.7–16.7)	10.4 (5.0-16.6)	-

*Friedmans test. SWE refers to shear wave elastography and ARFI to acoustic radiation force impulse. Advanced fibrosis is defined as a measurement of ≥ 9 kPa in the supine position using ARFI-SWE. Absence of advanced fibrosis is indicated by a measurement of < 9 kPa under the same conditions

### Factors associated with less reliable ARFI-SWE examinations

Univariate regression analysis revealed that, of the variables examined; sex, SAD, SCD, and cardiovascular medication use, only SAD and SCD were associated with less reliable ARFI-SWE examinations. Multivariate analysis of these significant factors further identified increased SAD to independently associate with less reliable ARFI-SWE examination (odds ratio [OR] = 1.20, *p* = 0.011; see Table 5).

Notably, sex-specific analyses demonstrated that higher SAD associated with less reliable ARFI-SWE examinations in women (OR = 1.33, *p* = 0.007), whereas SCD was associated to less reliable examinations in men (OR = 6.61, *p* = 0.009).

**Table 5:** Factors associated with less reliable ARFI-SWE examinations performed in supine

*N* = 200	β	*p*-value	Wald	Univariate OR	CI (95%)	β	*p*-value	Wald	Multiple OR	CI (95%)
**Sex**	0.10	0.806	0.06	1.11	0.49–2.48					
**Cardiovascular medication**	0.77	0.089	2.90	2.15	0.89–5.21					
women, *n* = 90	0.61	0.411	0.68	1.83	0.43–7.77					
men, *n* = 110	0.86	0.135	2.24	2.37	0.76–7.34					
**SAD** (cm)	0.27	< 0.001	23.37	1.31	1.17–1.46	0.18	0.011	6.41	1.20	1.04–6.59
women, *n* = 90	0.27	< 0.001	12.58	1.31	1.13–1.53	0.28	0.007	7.33	1.33	1.08–1.64
men, *n* = 110	0.26	< 0.001	10.97	1.30	1.11–1.52	0.10	0.293	1.11	1.11	0.92–1.34
**SCD** (cm)	1.76	< 0.001	22.22	5.78	2.79-12.00	0.92	0.063	3.47	2.51	0.95–6.59
women, *n* = 90	1.31	0.006	7.71	3.71	1.47–9.36	-0.13	0.860	0.03	0.88	0.21–3.72
men, *n* = 110	2.33	< 0.001	14.71	10.31	3.13–33.96	1.89	0.009	6.77	6.61	1.59–27.55

SAD refers to sagittal abdominal diameter, SCD to skin-to-liver capsule distance, SWE to shear wave elastography, ARFI to acoustic radiation force impulse, OR to odds ratio, and CI to confidence interval. In the multivariate analysis, the Nagelkerke R² was 27.6% and the Cox & Snell R² was 15.3%

## Discussion

In this investigation involving 200 patients diagnosed with chronic liver disease, ARFI-SWE examinations were performed across three different body positions (supine, 30° and 90° left decubitus), with SCD measurements also obtained in each position. The frequency of reliable examinations (defined reliable when inter-quartile range /median kPa was ≤ 30%) was compared across the three body positions. For subgroups characterized by increased SAD (cutoff ≥ 23 cm) and advanced fibrosis (cutoff  ≥ 9 kPa) comparisons were made regarding ARFI-SWE results, SCD values, and the number of reliable examinations, also stratified by sex. Factors associated with less reliable examinations were identified.

To the best of our knowledge, this study is the first to evaluate ARFI-SWE measurements in various body positions, also stratified for sex.

The main findings in this study revealed that SAD seems to have impact on ARFI-SWE results in patients with chronic liver disease. In patients with increased SAD, ARFI-SWE results did not vary between body positions; however, among those with normal SAD, ARFI-SWE values increased when transitioning from supine to left decubitus. SAD emerged as the sole determinant influencing examination reliability: for each centimeter increase in SAD, the risk of an unreliable examination rose by a factor of 1.2.

### Body position, ARFI-SWE results and SCD

In general ultrasound, fat droplets have lower sound speed and lower density than water, resulting in an impedance mismatch and therefore lipid vacuoles scatter ultrasound, creating a decreased visibility on ultrasound images [63]. A major challenge faced by many researchers and ARFI-SWE operators is the decreasing reliability of ARFI-SWE for obese patients with an increased SCD, which might be explained by several factors, including different influence on attenuation of ultrasound waves and push pulses in subcutaneous fat [64–66], shear wave scattering [67] and propagation [68], and the depth of the shear wave sample box in the liver [69]. Shear waves are affected by scattering, reflection, refraction, and anisotropic tissues resulting in estimation errors [70]. Push pulse transmission is affected by pulse strength, variations in tissue density, attenuation, absorption reflection, and scatter. The increased distance to the area of interest affects ultrasound waves attenuation and artefacts which altogether have a negative effect on the ultrasound image quality [71].

Therefore, to alter body position and increasing probe pressure are common techniques to obtain an acceptable B-mode image in abdominal ultrasound examinations. Moreover, increased probe pressure is found to decrease SCD and increase the number of technically successful measurements in ARFI-SWE examinations [49]. Our current findings indicate that more reliable ARFI-SWE results are achievable when SCD is decreased, which can be accomplished either by increasing probe pressure [49] or a postural change to left decubitus. However, previous studies involving healthy individuals have reported increased ARFI-SWE results in left decubitus [41; 42], indicating that this postural adjustment should be considered primarily in challenging cases. An earlier investigation of SCD with different ultrasound systems revealed differences in applicability, implying that different technologies can have different effects on ARFI-SWE results [46], and previous data demonstrate that ARFI-SWE comb-push technology is a reliable method for assessing liver fibrosis, especially in cases of increased SCD [72].

### Body position, liver stiffness and SAD

ARFI-SWE results in this study increased when altering body position from supine to left decubitus. However, our further sex-stratified analysis between each body position demonstrated no differences in ARFI-SWE results for men or women. Previous studies have noted the use of the 30° left decubitus position for patients during challenging ARFI-SWE examinations [73], and our findings confirm that this positioning is feasible without alter ARFI-SWE results.

Previous research in healthy subjects reported higher ARFI-SWE values in the left decubitus position compared to the supine position [43], which might indicate posture-related hemodynamic changes. Liver stiffness reportedly correlates with venous pressure [35], and the vena cava inferior diameter changes with different positions [74] and increased SAD also increases IAP [31]. Notably, SAD and IAP decrease following surgically induced weight loss [75]. Body position also reportedly influences blood flow variations in the liver, with an upright posture diminishing postprandial splanchnic hyperemia [40], and trans-jugular intrahepatic portosystemic shunt placement leads to decreases in both portal hypertension and shear wave speed [37, 76].

We utilized in our study a SAD cut-off of 23 cm to indicate increased risk, as previous research has identified a threshold of 22.7 cm for diagnosing metabolic syndrome and suggesting obesity [25]. The optimal SAD cut-off associated with an elevated calculated cardiometabolic risk score was found to be 22.2 cm for men and 20.1 cm for women [26].

In our study, SAD demonstrated a positive correlation with liver stiffness; specifically, each one-centimeter increase in SAD was associated with a corresponding increase in ARFI-SWE result. SAD might indicate liver disease, confirmed in a large health based study linked to liver related admissions, mortality and cancer, where SAD predicted incident liver disease, though weaker than waist-hip-ratio and waist-height ratio [28]. A longitudinal study involving 12,572 participants indicated that higher SAD values are linked to increased all-cause mortality risk [77]. Moreover, for liver fibrosis, SAD was independently associated with MASLD or significant fibrosis among non-obese subjects with MASLD [78].

The observed decrease in ARFI-SWE measurements among patients with advanced fibrosis, as opposed to the increase noted in those without advanced fibrosis when altering from supine to left decubitus position, may be attributed to differences in SAD between groups rather than the effects of fibrosis itself. Additionally, this reduction is likely indicative of posture-related hemodynamic changes rather than actual regression of fibrosis. It should also be noted that the sample size for the advanced fibrosis group was limited, and interpretations of these findings should be made with appropriate caution. Our findings may also have effects for shear wave propagation, respiratory mechanisms, and morphological changes of the liver across body positions, which may have impact on a repeated study resulting in different results.

### Body position and number of reliable examinations

Furthermore, our study demonstrated a significant increase in the number of reliable examinations when patients were positioned in the left decubitus posture, as compared to the supine position. Consequently, adopting the left decubitus position may enhance the efficiency of examinations. Variability in ARFI-SWE results may also be attributed to patient and/or operator movement, potentially impacting the accuracy of measurements [79]; therefore, it is essential for the ARFI-SWE operator to maintain a steady hand. Adjusting the patient’s posture to left decubitus enables the operator to rest their forearm on the patient’s body, which can be especially advantageous for individuals with obesity or fibrosis.

### Sex-based differences

In our study, ARFI-SWE results varied among women across different body positions, whereas no such variation was observed in men. Furthermore, subsequent sex-specific analyses did not reveal which specific body positions accounted for statistically significant differences, for either sex. These findings highlight the need for further research incorporating sex-based analysis to update guideline recommendations and improve diagnostic accuracy for both men and women. Earlier studies have confirmed higher liver stiffness results in men with shear wave elastography technologies [42, 54–56, 80], yet no studied has evaluated SAD and sex-based differences. A positive correlation between SAD and ARFI-SWE results were observed in both sexes. This association persisted among patients with elevated SAD; however, in the subgroup without increased SAD, no correlation was identified for either men or women. These findings suggest that SAD continues to be a noteworthy variable warranting further investigation in both men and women. Sex-based differences is also revealed as greater increase in risk for fibrosis among women compared to men which also highlights importance of sex-specific screening tools [81].

The factors contributing to less reliable examination in supine differed by sex, with increased SAD observed among women and SCD identified among men. This finding suggests that modifying body position could be an effective strategy to increase the success rate of ARFI-SWE examinations and decrease reliance on additional procedures such as liver biopsy or magnetic resonance elastography (MRE). In this study, we were unable to utilize MRI-PDFF for the assessment of hepatic steatosis, although MRI-PDFF is recognized as superior reliability than CAP [82]. As a result, steatosis was not included as a potential factor affecting examination reliability. Our primary aim was to investigate the impact of SAD and SCD on ARFI-SWE, and therefore steatosis was not prioritized in our analysis. Additionally, BMI is acknowledged as an imprecise indicator of body fat, since individuals categorized as overweight based on BMI may be healthy by other physical and physiological measures and do not necessarily exhibit obesity [83]. Consequently, BMI was excluded from the anthropometric measurements considered as possible contributors to less reliable examinations.

### Clinical implications

In accordance with EFSUMB guidelines [11] and the recently published WFUMB guidelines [61] evaluation of liver fibrosis using shear wave elastography techniques is recommended to be conducted with the patient in the supine position. Given the rising prevalence of MASLD, obesity, and overweight globally, there is likely to be an increase in complex cases and difficulties to obtaining reliable ARFI-SWE examinations. For patients with increased SAD, adjusting the body position to left decubitus may provide a superior acoustic B-mode window. This approach appears feasible for both men and women with chronic liver disease and does not result in elevated ARFI-SWE values.

### Strengths and limitations of the study

A key strength of this study is that all participants underwent examination and measurement on the same day using a consistent ultrasound device and operator. Additionally, the sample included an equal number of men and women, which further contributes to the robustness of the research design.

There are certain limitations to our study. The absence of a reference method for assessing liver fibrosis represents a constraint, underscoring the necessity for validation of our results using MRE as a reference method. Furthermore, a comprehensive assessment of hemodynamic effects on liver stiffness across various body positions with measurement and analysis of flow variations in the vena cava, portal vein, and abdominal aorta would further develop the scientific evaluation of the impact of different body positions on liver stiffness measurement. Variations in the SAD cutoff values applied during analysis could yield different outcomes; therefore, further investigations incorporating diverse SAD cutoffs are warranted, as a definitive SAD cutoff for liver diseases has yet to be established.

The generalizability of our findings must however be discussed since data in our prospective study was collected in 2017–2018. The GE LOGIQ shear wave elastography comb-push technology has largely remained unchanged since 2017, including its use of the same frequency and the machine specific cutoff has not changed since 2017. However, improvements have been made to the underlying B-mode image and the software, and we concur that these enhancements may positively impact ARFI-SWE reliability. In this study, we used the new cutoff for advanced fibrosis (≥ 9 kPa) according to WFUMB 2024 guidelines, and not the machine specific cutoff for advanced fibrosis. Moreover, the difference between EFSUMB guidelines from 2017 and WFUMB guidelines from 2024 has not changed the clinical performance of ARFI-SWE measurements. Therefore, our findings might still be relevant.

Additionally, this research was conducted at a single site by a single operator, which may affect the reproducibility of the findings. The findings from this study, including observed sex-based differences, warrant evaluation in cohorts with varying disease etiologies and further validation in larger multi-center and multi-vendor cohorts. Establishing more individualized thresholds and assessing performance according to sex, ethnicity, and all stages of fibrosis is essential. Additionally, potential sex-related impacts on results should be carefully examined to minimize the risk of missed diagnoses in any group.

### In conclusion

Our main findings suggest that, with higher SAD, repositioning patients to the left decubitus can enhance the number of reliable examinations and reduce SCD without elevating ARFI-SWE results, irrespectively of sex. SAD seems to have an impact on ARFI-SWE results, and correlates to liver stiffness. Increased SAD emerged as the sole determinant with negative impact on examination reliability.

## Data Availability

Data and materials supporting the results or analyses presented in this paper will be made available upon reasonable request.

## Abbreviations

ARFI: Acoustic radiation force impulse
ROI: Region of interest
SAD: Sagittal abdominal diameter
SCD: Skin-to liver capsule distance
SWE: Shear wave elastography

## References

[1] EASL. EASL recommendations on treatment of hepatitis C: Final update of the series(☆). J Hepatol. 2020;73:1170–218. 32956768 10.1016/j.jhep.2020.08.018

[2] EASL. EASL Clinical Practice Guidelines: Autoimmune hepatitis. J Hepatol. 2015;63:971–1004. 26341719 10.1016/j.jhep.2015.06.030

[3] EASL. EASL 2017 Clinical Practice Guidelines on the management of hepatitis B virus infection. J Hepatol. 2017. 10.1016/j.jhep.2017.03.021. 10.1016/j.jhep.2017.03.021 28427875

[4] EASL. EASL Clinical Practice Guidelines: Management of alcohol-related liver disease. J Hepatol. 2018;69:154–81. 29628280 10.1016/j.jhep.2018.03.018

[5] EASL. EASL-EASD-EASO Clinical Practice Guidelines on the management of metabolic dysfunction-associated steatotic liver disease (MASLD). J Hepatol. 2024;81:492–542. 38851997 10.1016/j.jhep.2024.04.031

[6] Younossi ZM, Wong G, Anstee QM, Henry L. The Global Burden of Liver Disease. Clin Gastroenterol Hepatol. 2023. 10.1016/j.cgh.2023.04.015. 37121527 10.1016/j.cgh.2023.04.015

[7] EASL. EASL Clinical Practice Guidelines on non-invasive tests for evaluation of liver disease severity and prognosis – 2021 update. J Hepatol. 2021;75:659–89. 34166721 10.1016/j.jhep.2021.05.025

[8] Gines P, Quintero E, Arroyo V, et al. Compensated cirrhosis: natural history and prognostic factors. Hepatology. 1987;7:122–8. 3804191 10.1002/hep.1840070124

[9] Herrmann E, de Ledinghen V, Cassinotto C, et al. Assessment of biopsy-proven liver fibrosis by two-dimensional shear wave elastography: An individual patient data-based meta-analysis. Hepatology. 2018;67:260–72. 28370257 10.1002/hep.29179 PMC5765493

[10] Bota S, Herkner H, Sporea I, et al. Meta-analysis: ARFI elastography versus transient elastography for the evaluation of liver fibrosis. Liver Int. 2013;33:1138–47. 23859217 10.1111/liv.12240

[11] Dietrich CF, Bamber J, Berzigotti A, et al. EFSUMB Guidelines and Recommendations on the Clinical Use of Liver Ultrasound Elastography, Update 2017 (Long Version). Ultraschall Med. 2017;38:e16–47. 28407655 10.1055/s-0043-103952

[12] Ferraioli G, Wong VW, Castera L, et al. Liver Ultrasound Elastography: An Update to the World Federation for Ultrasound in Medicine and Biology Guidelines and Recommendations. Ultrasound Med Biol. 2018;44:2419–40. 30209008 10.1016/j.ultrasmedbio.2018.07.008

[13] Ferraioli G, De Silvestri A, Reiberger T, et al. Adherence to quality criteria improves concordance between transient elastography and ElastPQ. for liver stiffness assessment — a multicenter retrospective study; 2018. 10.1016/j.dld.2018.03.033 29705030

[14] Boursier J, Cassinotto C, Hunault G, et al. Criteria to Determine Reliability of Noninvasive Assessment of Liver Fibrosis With Virtual Touch Quantification. Clin Gastroenterol Hepatol. 2019;17:164–e171165. 29753082 10.1016/j.cgh.2018.04.062

[15] Kollmann C, Jenderka KV, Moran CM, Draghi F, Jimenez Diaz JF, Sande R. EFSUMB Clinical Safety Statement for Diagnostic Ultrasound – (2019 revision). Ultraschall Med. 2020;41:387–9. 31594007 10.1055/a-1010-6018

[16] Byenfeldt M, Both S, Bazzi M, Wallin A. Radiographers’ perspective of patient safety at ultrasound units in radiology departments. Radiography (Lond). 2025;31:152–8. 39571263 10.1016/j.radi.2024.11.006

[17] Bota S, Sporea I, Sirli R, et al. Factors associated with the impossibility to obtain reliable liver stiffness measurements by means of Acoustic Radiation Force Impulse (ARFI) elastography–analysis of a cohort of 1,031 subjects. Eur J Radiol. 2014;83:268–72. 24360231 10.1016/j.ejrad.2013.11.019

[18] Bruce M, Kolokythas O, Ferraioli G, Filice C, O’Donnell M. Limitations and artifacts in shear-wave elastography of the liver. Biomed Eng Lett. 2017;7:81–9. 30603154 10.1007/s13534-017-0028-1 PMC6208474

[19] Castera L, Foucher J, Bernard PH, et al. Pitfalls of liver stiffness measurement: a 5-year prospective study of 13,369 examinations. Hepatology. 2010;51:828–35. 20063276 10.1002/hep.23425

[20] Dietrich CF, Shi L, Wei Q, et al. What does liver elastography measure? Technical aspects and methodology. Minerva Gastroenterol Dietol. 2020. 10.23736/s1121-421x.20.02787-7. 10.23736/S2724-5985.20.02787-7 33267564

[21] Guglielmo FF, Barr RG, Yokoo T, et al. Liver Fibrosis, Fat, and Iron Evaluation with MRI and Fibrosis and Fat Evaluation with US: A Practical Guide for Radiologists. Radiographics. 2023;43:e220181. 37227944 10.1148/rg.220181

[22] Urban M, Vasconcelos L, Brom K, Dave J, Kijanka P. Shear wave elastography primer for the abdominal radiologist. Abdom Radiol (NY). 2025. 10.1007/s00261-025-04806-1. 39883164 10.1007/s00261-025-04806-1 PMC12266967

[23] WHO. (2025) Obesity. (Internet). World Health Organization. Available via https://www.who.int. Accessed 2026-01-29.

[24] Fox CS, Massaro JM, Hoffmann U, et al. Abdominal visceral and subcutaneous adipose tissue compartments: association with metabolic risk factors in the Framingham Heart Study. Circulation. 2007;116:39–48. 17576866 10.1161/CIRCULATIONAHA.106.675355

[25] Hoenig MR. MRI sagittal abdominal diameter is a stronger predictor of metabolic syndrome than visceral fat area or waist circumference in a high-risk vascular cohort. Vasc Health Risk Manag. 2010;6:629–33. 20730019 10.2147/vhrm.s10787 PMC2922324

[26] Riserus U, de Faire U, Berglund L, Hellenius ML. (2010) Sagittal abdominal diameter as a screening tool in clinical research: cutoffs for cardiometabolic risk. Journal of Obesity 2010. 10.1155/2010/757939 PMC2925288 20798888

[27] Kahn HS. Replacing the body mass index with the sagittal abdominal diameter (abdominal height). Obes (Silver Spring). 2023;31:2720–2. 10.1002/oby.23889 37749805

[28] Aberg F, Jula A. The sagittal abdominal diameter: Role in predicting severe liver disease in the general population. Obes Res Clin Pract. 2018;12:394–6. 30078405 10.1016/j.orcp.2018.06.007

[29] Varela JE, Hinojosa M, Nguyen N. Correlations between intra-abdominal pressure and obesity-related co-morbidities. Surg Obes Relat Dis. 2009;5:524–8. 19560978 10.1016/j.soard.2009.04.003

[30] Nordhamn K, Sodergren E, Olsson E, Karlstrom B, Vessby B, Berglund L. Reliability of anthropometric measurements in overweight and lean subjects: consequences for correlations between anthropometric and other variables. Int J Obes Relat Metab Disord. 2000;24:652–7. 10849590 10.1038/sj.ijo.0801216

[31] Sugerman H, Windsor A, Bessos M, Wolfe L. Intra-abdominal pressure, sagittal abdominal diameter and obesity comorbidity. J Intern Med. 1997;241:71–9. 9042096 10.1046/j.1365-2796.1997.89104000.x

[32] Kadoya Y, Miyati T, Kobayashi S, Ohno N, Gabata T. Evaluation of gravity effect on inferior vena cava and abdominal aortic flow using multi-posture MRI. Acta Radiol. 2021;62:1122–8. 32799558 10.1177/0284185120950112

[33] Ishizaki Y, Fukuoka H, Ishizaki T, et al. Measurement of inferior vena cava diameter for evaluation of venous return in subjects on day 10 of a bed-rest experiment. J Appl Physiol (1985). 2004;96:2179–86. 14990554 10.1152/japplphysiol.01097.2003

[34] Ohnishi K, Saito M, Nakayama T, et al. Portal venous hemodynamics in chronic liver disease: effects of posture change and exercise. Radiology. 1985;155:757–61. 3890004 10.1148/radiology.155.3.3890004

[35] Millonig G, Friedrich S, Adolf S, et al. Liver stiffness is directly influenced by central venous pressure. J Hepatol. 2010;52:206–10. 20022130 10.1016/j.jhep.2009.11.018

[36] Piecha F, Peccerella T, Bruckner T, Seitz HK, Rausch V, Mueller S. Arterial pressure suffices to increase liver stiffness. Am J Physiology: Gastrointest Liver Physiol. 2016;311:G945–53. 10.1152/ajpgi.00399.2015 27288426

[37] Piecha F, Paech D, Sollors J, et al. Rapid change of liver stiffness after variceal ligation and TIPS implantation. Am J Physiology: Gastrointest Liver Physiol. 2018;314:G179–87. 10.1152/ajpgi.00239.2017 29051188

[38] Reiberger T, Ferlitsch A, Payer BA, Pinter M, Homoncik M, Peck-Radosavljevic M. Non-selective beta-blockers improve the correlation of liver stiffness and portal pressure in advanced cirrhosis. J Gastroenterol. 2012;47:561–8. 22170417 10.1007/s00535-011-0517-4

[39] Goertz RS, Egger C, Neurath MF, Strobel D. Impact of food intake, ultrasound transducer, breathing maneuvers and body position on acoustic radiation force impulse (ARFI) elastometry of the liver. Ultraschall Med. 2012;33:380–5. 22723037 10.1055/s-0032-1312816

[40] Iwao T, Oho K, Nakano R, et al. Upright posture blunts postprandial splanchnic hyperemia in patients with cirrhosis and portal hypertension. J Gastroenterol. 1999;34:359–65. 10433012 10.1007/s005350050274

[41] Huang Z, Zheng J, Zeng J, Wang X, Wu T, Zheng R. Normal liver stiffness in healthy adults assessed by real-time shear wave elastography and factors that influence this method. Ultrasound Med Biol. 2014;40:2549–55. 25282481 10.1016/j.ultrasmedbio.2014.05.008

[42] Liao LY, Kuo KL, Chiang HS, Lin CZ, Lin YP, Lin CL. Acoustic radiation force impulse elastography of the liver in healthy patients: test location, reference range and influence of gender and body mass index. Ultrasound Med Biol. 2015;41:698–704. 25638317 10.1016/j.ultrasmedbio.2014.09.030

[43] Jiang X, Li L, Xue HY. The impact of body position and exercise on the measurement of liver Young’s modulus by real-time shear wave elastography. Technol Health Care. 2022;30:445–54. 34657862 10.3233/THC-213218

[44] Uchikawa S, Kawaoka T, Fujino H, et al. The effect of the skin-liver capsule distance on the accuracy of ultrasound diagnosis for liver steatosis and fibrosis. J Med Ultrason (2001). 2022;49:443–50. 35524897 10.1007/s10396-022-01210-w

[45] Staugaard B, Christensen PB, Mössner B, et al. Feasibility of transient elastography versus real-time two-dimensional shear wave elastography in difficult-to-scan patients. Scand J Gastroenterol. 2016;51:1354–9. 27310486 10.1080/00365521.2016.1193217

[46] Lee SM, Chang W, Kang HJ, Ahn SJ, Lee JH, Lee JM. Comparison of four different Shear Wave Elastography platforms according to abdominal wall thickness in liver fibrosis evaluation: a phantom study. Med Ultrasonography. 2019;21:22–9. 10.11152/mu-1737 30779827

[47] Giuffrè M, Giuricin M, Bonazza D et al. (2020) Optimization of Point-Shear Wave Elastography by Skin-to-Liver Distance to Assess Liver Fibrosis in Patients Undergoing Bariatric Surgery. Diagnostics (Basel) 10. 10.3390/diagnostics10100795 PMC7601552 33036418

[48] Byenfeldt M, Elvin A, Fransson P. On Patient Related Factors and Their Impact on Ultrasound-Based Shear Wave Elastography of the Liver. Ultrasound Med Biol. 2018;44:1606–15. 29735314 10.1016/j.ultrasmedbio.2018.03.031

[49] Byenfeldt M, Elvin A, Fransson P. Influence of Probe Pressure on Ultrasound-Based Shear Wave Elastography of the Liver Using Comb-Push 2-D Technology. Ultrasound Med Biol. 2019;45:411–28. 30401508 10.1016/j.ultrasmedbio.2018.09.023

[50] Wirth A, Steinmetz B. Gender differences in changes in subcutaneous and intra-abdominal fat during weight reduction: an ultrasound study. Obes Res. 1998;6:393–9. 9845228 10.1002/j.1550-8528.1998.tb00370.x

[51] Kuk JL, Lee S, Heymsfield SB, Ross R. Waist circumference and abdominal adipose tissue distribution: influence of age and sex. Am J Clin Nutr. 2005;81:1330–4. 15941883 10.1093/ajcn/81.6.1330

[52] Carlsson AC, Riserus U, Arnlov J, et al. Prediction of cardiovascular disease by abdominal obesity measures is dependent on body weight and sex–results from two community based cohort studies. Nutr Metabolism Cardiovasc Dis. 2014;24:891–9. 10.1016/j.numecd.2014.02.001 24680224

[53] Halaoui AF, Ali AH, Habib SG, et al. Gender differences in liver fibrosis among patients younger than 50 years: A retrospective cohort study. Clin Res Hepatol Gastroenterol. 2020;44:733–8. 32169461 10.1016/j.clinre.2020.01.001

[54] Mulabecirovic A, Mjelle AB, Gilja OH, Vesterhus M, Havre RF. Liver elasticity in healthy individuals by two novel shear-wave elastography systems-Comparison by age, gender, BMI and number of measurements. PLoS ONE. 2018;13:e0203486. 30216377 10.1371/journal.pone.0203486 PMC6138384

[55] Roulot D, Czernichow S, Le Clésiau H, Costes J-L, Vergnaud A-C, Beaugrand M. Liver stiffness values in apparently healthy subjects: Influence of gender and metabolic syndrome. J Hepatol. 2008;48:606–13. 18222014 10.1016/j.jhep.2007.11.020

[56] Corpechot C, El Naggar A, Poupon R. Gender and liver: is the liver stiffness weaker in weaker sex? Hepatology. 2006;44:513–4. 16871581 10.1002/hep.21306

[57] Bissell MD. (1999) Sex and hepatic fibrosis. LWW, pp 988–989. 10.1002/hep.510290351 10051508

[58] Crudele L, De Matteis C, Novielli F, et al. Fatty Liver Index (FLI) is the best score to predict MASLD with 50% lower cut-off value in women than in men. Biology Sex Differences. 2024;15:43. 10.1186/s13293-024-00617-z PMC11100212 38760802

[59] Milani I, Parrotta ME, Colangeli L et al. (2025) Sex Differences in MASLD After Age 50: Presentation, Diagnosis, and Clinical Implications. Biomedicines 13. 10.3390/biomedicines13092292 PMC12467267 41007852

[60] Karlas T, Petroff D, Sasso M, et al. Individual patient data meta-analysis of controlled attenuation parameter (CAP) technology for assessing steatosis. J Hepatol. 2016. 10.1016/j.jhep.2016.12.022. 28039099 10.1016/j.jhep.2016.12.022

[61] Ferraioli G, Barr RG, Berzigotti A, et al. WFUMB Guideline/Guidance on Liver Multiparametric Ultrasound: Part 1. Update to 2018 Guidelines on Liver Ultrasound Elastography. Ultrasound Med Biol. 2024;50:1071–87. 38762390 10.1016/j.ultrasmedbio.2024.03.013

[62] Bland JM, Altman DG. Applying the right statistics: analyses of measurement studies. Ultrasound Obstet Gynecol. 2003;22:85–93. 12858311 10.1002/uog.122

[63] Shung KK, Thieme GA. Ultrasonic scattering in biological tissues. CRC; 2022.

[64] Mast TD, Hinkelman LM, Metlay LA, Orr MJ, Waag RC. Simulation of ultrasonic pulse propagation, distortion, and attenuation in the human chest wall. J Acoust Soc Am. 1999;106:3665–77. 10615705 10.1121/1.428209

[65] Barry CT, Mills B, Hah Z, et al. Shear wave dispersion measures liver steatosis. Ultrasound Med Biol. 2012;38:175–82. 22178165 10.1016/j.ultrasmedbio.2011.10.019 PMC3428716

[66] Dahl JJ, Sheth NM. Reverberation clutter from subcutaneous tissue layers: simulation and in vivo demonstrations. Ultrasound Med Biol. 2014;40:714–26. 24530261 10.1016/j.ultrasmedbio.2013.11.029 PMC3942094

[67] Wang Y, Jiang J. Influence of Tissue Microstructure on Shear Wave Speed Measurements in Plane Shear Wave Elastography: A Computational Study in Lossless Fibrotic Liver Media. Ultrason Imaging. 2018;40:49–63. 28720056 10.1177/0161734617719055

[68] Guo YR, Chen X, Lin H, Zhang X. (2013) In-vitro quantification of rat liver viscoelasticity with shear wave dispersion ultrasound vibrometry. Conference Proceedings of the Annual International Conference of the IEEE Engineering in Medicine and Biology Society 2013:1915–1918. 10.1109/EMBC.2013.6609900 24110087

[69] Zhao H, Song P, Urban MW, et al. Bias observed in time-of-flight shear wave speed measurements using radiation force of a focused ultrasound beam. Ultrasound Med Biol. 2011;37:1884–92. 21924817 10.1016/j.ultrasmedbio.2011.07.012 PMC3199321

[70] O’Hara S, Zelesco M, Rocke K, Stevenson G, Sun Z. (2019) Reliability Indicators for 2-Dimensional Shear Wave Elastography. Journal of Ultrasound in Medicine 0. 10.1002/jum.14984 30887548

[71] Caraiani C, Dong Y, Rudd AG, Dietrich CF. (2018) Reasons for inadequate or incomplete imaging techniques. 2018. 10.11152/mu-1736 10.11152/mu-1736 30534659

[72] Serra C, Grasso V, Conti F, et al. A New Two-Dimensional Shear Wave Elastography for Noninvasive Assessment of Liver Fibrosis in Healthy Subjects and in Patients with Chronic Liver Disease. Ultraschall Med. 2018;39:432–9. 29458217 10.1055/s-0043-119356

[73] Cui XW, Friedrich-Rust M, De Molo C, Ignee A, Schreiber-Dietrich D, Dietrich CF. Liver elastography, comments on EFSUMB elastography guidelines 2013. World J Gastroenterol. 2013;19:6329–47. 24151351 10.3748/wjg.v19.i38.6329 PMC3801303

[74] Kundra P, Arunsekar G, Vasudevan A, Vinayagam S, Habeebullah S, Ramesh A. Effect of postural changes on inferior vena cava dimensions and its influence on haemodynamics during caesarean section under spinal anaesthesia. J Obstet Gynaecol. 2015;35:667–71. 25546523 10.3109/01443615.2014.991291

[75] Sugerman H, Windsor A, Bessos M, Kellum J, Reines H, DeMaria E. Effects of surgically induced weight loss on urinary bladder pressure, sagittal abdominal diameter and obesity co-morbidity. Int J Obes Relat Metab Disord. 1998;22:230–5. 9539191 10.1038/sj.ijo.0800574

[76] Tzschatzsch H, Sack I, Marticorena Garcia SR, et al. Time-Harmonic Elastography of the Liver is Sensitive to Intrahepatic Pressure Gradient and Liver Decompression after Transjugular Intrahepatic Portosystemic Shunt (TIPS) Implantation. Ultrasound Med Biol. 2017;43:595–600. 27979668 10.1016/j.ultrasmedbio.2016.10.007

[77] Gu X, Gao P, Zhu F, Shen Y, Lu L. Association between sagittal abdominal diameter-to-height ratio and all-cause mortality among adults in the United States: a longitudinal study. Arch Public Health. 2024;82:213. 39538327 10.1186/s13690-024-01443-w PMC11562676

[78] Kim D, Kim W, Joo SK, et al. Predictors of nonalcoholic steatohepatitis and significant fibrosis in non-obese nonalcoholic fatty liver disease. Liver Int. 2019;39:332–41. 30298568 10.1111/liv.13983

[79] Shin HJ, Kim MJ, Yoon CS, et al. Motion effects on the measurement of stiffness on ultrasound shear wave elastography: a moving liver fibrosis phantom study. Med Ultrasonography. 2018;1:14–20. 10.11152/mu-1138 29400362

[80] Colombo S, Belloli L, Zaccanelli M, et al. Normal liver stiffness and its determinants in healthy blood donors. Dig Liver Disease. 2011;43:231–6. 10.1016/j.dld.2010.07.008 20817625

[81] Albhaisi S, Kim S, Terrault N, Dodge JL. Sex-Specific Cardiometabolic Profiles and Severity of Liver Fibrosis. JAMA Netw Open. 2026;9:e260863. 41801201 10.1001/jamanetworkopen.2026.0863 PMC12973100

[82] Ferraioli G, Barr RG, Berzigotti A, et al. WFUMB Guidelines/Guidance on Liver Multiparametric Ultrasound. Part 2: Guidance on Liver Fat Quantification. Ultrasound Med Biol. 2024;50:1088–98. 38658207 10.1016/j.ultrasmedbio.2024.03.014

[83] Rodrigues IG, Arcoverde G, do Nascimento CLC, et al. Sex-Specific Differences in Visceral and Subcutaneous Adiposity Accumulation and Their Association With Metabolic Abnormalities. J Obes. 2025;2025:7240063. 41189625 10.1155/jobe/7240063 PMC12582646

